# Advantage of Hole Stimulus in Rivalry Competition

**DOI:** 10.1371/journal.pone.0033053

**Published:** 2012-03-22

**Authors:** Qianli Meng, Ding Cui, Ke Zhou, Lin Chen, Yuanye Ma

**Affiliations:** 1 Laboratory of Primate Cognitive Neuroscience, Kunming Institute of Zoology, Chinese Academy of Sciences, Kunming, Yunnan, China; 2 Graduate University of Chinese Academy of Science, Beijing, China; 3 State Key Laboratory of Brain and Cognitive Science, Institute of Biophysics, Chinese Academy of Sciences, Beijing, China; Cuban Neuroscience Center, Cuba

## Abstract

Mounting psychophysical evidence suggests that early visual computations are sensitive to the topological properties of stimuli, such as the determination of whether the object has a hole or not. Previous studies have demonstrated that the hole feature took some advantages during conscious perception. In this study, we investigate whether there exists a privileged processing for hole stimuli during unconscious perception. By applying a continuous flash suppression paradigm, the target was gradually introduced to one eye to compete against a flashed full contrast Mondrian pattern which was presented to the other eye. This method ensured that the target image was suppressed during the initial perceptual period. We compared the initial suppressed duration between the stimuli with and without the hole feature and found that hole stimuli required less time than no-hole stimuli to gain dominance against the identical suppression noise. These results suggest the hole feature could be processed in the absence of awareness, and there exists a privileged detection of hole stimuli during suppressed phase in the interocular rivalry.

## Introduction

According to the “Global-first” topological approach to visual perception [1]–[3], the first step in object representation is the extraction of topological properties, particularly the determination of whether the object has a hole or not. Further, other studies have shown that the presence of closure enjoys some advantages during conscious visual perception, suggesting that closure can be rapidly recognized by the visual system as a simple or primitive property [4]–[9]. For instance, Elder and Zucker found that two dimensional shape processing is rapid for the closed stimuli but slow for the open stimuli [6], . However, it remains unclear whether there exists a privileged processing for hole stimuli during unconscious perception.

It should be mentioned that the concept of a “hole” in the present study speaks to a two-dimensional concept, which does not require any extended surface, or figure-ground structure. In this sense, the concept of a “hole” in the present is same as the concept of “closure” in the gestalt tradition [4], [6], [7]. Thus, our definition of the “hole” is fundamentally different from that defined in previous studies on “hole” perception, in which the “hole” is defined as a background region that are surrounded by a foreground figure [10]–[13].

Recently, continuous flash suppression (CFS) [14], [15], a particularly potent variant of binocular rivalry to render stimuli presented to one eye invisible for many seconds at a time, has been proved to be an optimal technique to investigate the degree to which invisible stimuli are processed in the absence of conscious awareness. Unconscious processing can be inferred from the time that initially invisible stimuli need to overcome the suppression noise and become dominant. For instance, by using this breaking CFS paradigm, Jiang *et al*., has found an enhanced unconscious processing for familiar and recognizable stimuli, as evidenced by the shorter suppression durations for upright faces compared with upside-down faces [16].

In the present study, we used the breaking CFS paradigm to investigate the difference in processing between the hole and no-hole feature in the absence of visual awareness. At the beginning of each trial, a flashed full contrast Mondrian pattern noise was presented to the subject's dominant eye, and the target image was gradually introduced to the other eye. This method ensured that at the start of the trial, the image that subjects perceived was the noise, not the target. We compared the initial suppressed duration between the stimuli with and without hole feature.

## Methods

### Ethics statement

Both experiments were performed according to the principles expressed in the Declaration of Helsinki and had approval from the Human Research Ethics Committee of the Institute of Biophysics, Chinese Academy of Sciences. All participants provided written informed consent for the collection of data and subsequent analysis.

### Subjects

Sixteen undergraduate students (8 males) were paid to participate in Experiment 1, and twenty undergraduate students (11 males) were paid to participate in Experiment 2. All the subjects were 21 to 29 years of age and had normal vision except for a corrected mild myopia. All the subjects were recruited in Beijing universities by advertisement and took part in the experiments voluntarily. They were all right-handed.

### Stimuli and Procedures

**Figure 1:**
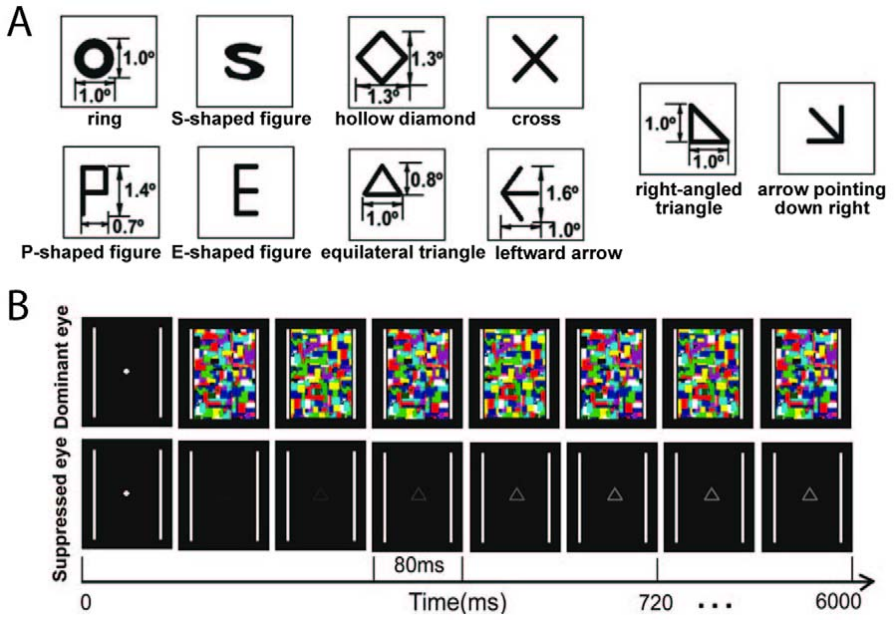
Stimuli and Procedures. (A) Schematic depiction of the stimulus pairs. (B) Schematic representation of the binocular rivalry paradigm.

As shown in Fig. 1A, the target images consisted of two groups of figures: one with hole feature (ring, P-shaped figure, hollow diamond, equilateral triangle and right-angled triangle) and one without hole feature (S-shaped figure, E-shaped figure, cross, leftward arrow and arrow pointing down right). To rule out confounds based on local features, we carefully designed the stimuli to minimize the difference in local features of many of the low-level physical properties between the “hole” and “no-hole” stimuli. The ring and S-shaped figure (hereafter referred to as S) were designed to have equal luminous flux and nearly equal spatial frequency components and perimeter lengths as well as equal average edge crossings [1], [2]. P-shaped figure and E-shaped figure (hereafter respectively referred to as P and E) were composed of equal numbers and lengths of line segments [2]. Line segments of equal lengths and identical orientations were present in the members of the following pairs: hollow diamond vs. cross, equilateral triangle vs. leftward arrow, and right-angled triangle vs. arrow pointing down right [17].

The stimuli were presented with MATLAB using the Psychophysics Toolbox on a 19-in ViewSonic monitor (100 Hz). The images were fused using a mirror stereoscope mounted on a chin rest. A frame (10.5^0^×9.5^0^) extended beyond the outer border of the noise. The viewing distance was 81 cm. A fixation cross (0.4^0^×0.4^0^) was presented to each eye before the start of the trial. At the beginning of each trial, a full contrast chromatic Mondrian pattern (which comprised twelve differently colored elements) flashing at 10 Hz was presented to the observer's dominant eye. To prevent the subject from fixing on the same area on which the target image would appear, the target images were gradually presented to the non-dominant eye at one of the five locations within the region corresponding to the location of the noise: at the center of the suppressed field or 1° above, below, to the left, or to the right of the center of the suppressed field. The luminance contrast of the target image summed up with 6% speed from 2% to 50% during the initial 0.72 s of the trial, after which time it remained constant until the subject pressed the button to stop the trial (for which there was a time limit of 6 s) (Fig. 1B). There were 75 trials for each condition (hole and no-hole), 15 trials for each type of stimuli (ring, S, P, E, cross, hollow diamond, equilateral triangle, leftward arrow, right-angled triangle and arrow pointing down right). To diminish possible response biases, 9% (15) catch trials in which no target image were presented were included in the study. The stimuli were presented in a randomized sequence.

In Experiment 1, the subjects were asked to detect the appearance of the target image as rapidly as possible and to report whether it was a “hole” or a “no-hole” stimulus. Once the target image was discriminated, the subject pressed a button to stop the trial, and the reaction time was recorded.

**Figure 2:**
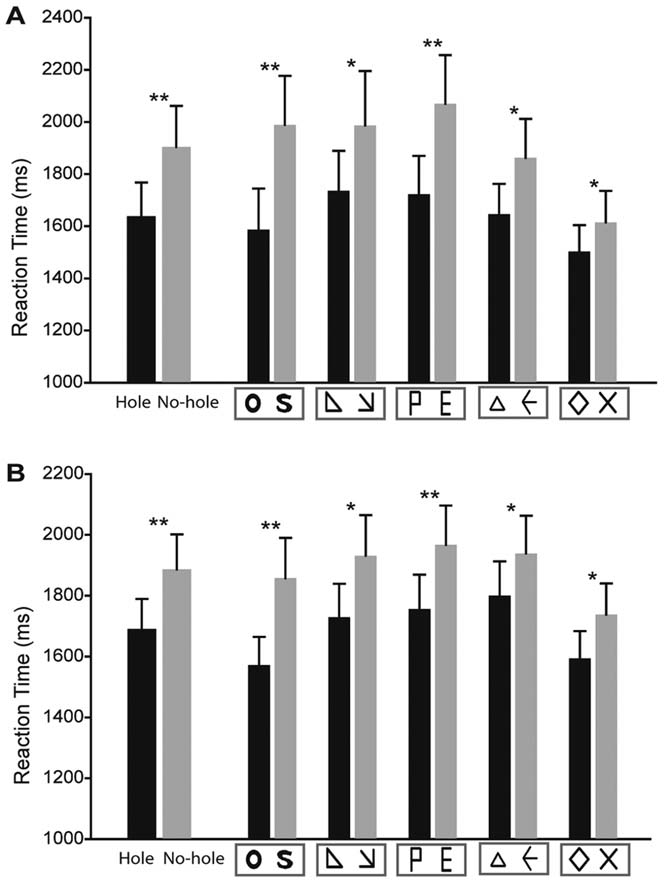
Results of Exp 1 and 2. (A) The average reaction times for the “hole” and “no-hole” stimuli in Experiment 1. (B) The average reaction times for the “hole” and “no-hole” stimuli in Experiment 2. * p<0.01; ** p<0.002.

Because the task was to discern whether the target image contained a hole, it might be difficult to determine whether the suppression was composed of two phases, in which the subject needed to both detect the presence of a stimulus and determine what type of target image was presented. Thus, once the subject became aware of the target image, he or she needed additional time to identify the target image. To exclude this additional phase of stimulus identification, the subjects merely performed a simple detection task in Experiment 2. In each trial, as soon as the subjects detected the target image or any part of them, the trial stopped. This practice ensured that the suppression duration was based solely on the time the target image remained invisible.

The “Z” and “M” keys on a computer keyboard were used indicate “hole” and “no-hole” in Experiment 1 and “yes” and “no” in Experiment 2.

## Results

The mean false-alarm rates on catch trials were 2% for Experiment 1 and zero for Experiment 2. For each type of target image (ring, S, P, E, cross, hollow diamond, equilateral triangle, leftward arrow, right-angled triangle and arrow pointing down right), data were pooled across five locations since there are only 3 trials in each location. The mean accuracies and reaction times (RTs) were analyzed using a paired T-test with Bonferroni correction, respectively [18]. 0.05 was chosen as the significant level and divided it by the no. of pairwise comparisons (5[: ring vs. S, P vs. E, hollow diamond vs. cross, equilateral triangle vs. leftward arrow, right-angled triangle vs. arrow pointing down right]). A significant level of 0.01 was yielded.

### Experiment 1: Discrimination task

First, all types of hole stimuli (ring, P, hollow diamond, equilateral triangle, and right-angled triangle) were grouped as one hole category, and all no-hole stimuli (S, E, cross, leftward arrow, and arrow pointing down right) were grouped as no-hole category. The mean accuracy for the hole category were significantly higher than that of the no-hole category (t [15] = 2.35, p = 0.03). Then, we conducted separate comparisons for each stimulus-pair. The accuracy of the ring is slightly higher than that of S (t [15] = 2.71, p = 0.016). No significant differences were found in all the other stimulus-pair comparisons (right-angled triangle vs. arrow pointing down right, t [15] = −0.487, p = 0.63; P vs. E, t [15] = 1.00, p = 0.33; equilateral triangle vs. leftward arrow, t [15] = 2.05, p = 0.054; hollow diamond vs. cross, t [15] = 0.70, p = 0.50).

As shown in Fig. 2A, the mean RT of the hole category was significantly shorter than that of the no-hole category (t [15] = −6.22, p<0.001). Similar results were found when conducting separate comparisons for each well-controlled stimulus-pair (ring vs. S, t [15] = −5.19, p<0.001; right-angled triangle vs. arrow pointing down right, t [15] = −2.83, p = 0.01; P vs. E, t [15] = −3.73, p = 0.002; equilateral triangle vs. leftward arrow, t [15] = −2.92, p = 0.01; hollow diamond vs. cross, t [15] = −2.93, p = 0.01).

### Experiment 2: Detection task

No significant difference was observed between the mean accuracies of the hole and no-hole categories (t [19] = 1.48, p = 0.15). And there was no significant difference in each stimulus-pair comparison (ring vs. S, t [19] = 2.13, p = 0.05; right-angled triangle vs. arrow pointing down right, t [19] = −0.36, p = 0.72; P vs. E, t [19] = 1.44, p = 0.17; equilateral triangle vs. leftward arrow, t [19] = 0.18, p = 0.86; hollow diamond vs. cross, t [19] = 0.83, p = 0.43).

As illustrated in Fig. 2B, figures in the hole category were detected more rapidly as compared with those in the no-hole category during the suppressed phase (t [19] = −5.74, p<0.001). Moreover, we found similar results in each stimulus-pair comparison (ring vs. S, t [19] = −3.81, p = 0.001; right-angled triangle vs. arrow pointing down right, t [19] = −2.71, p = 0.01; P vs. E, t [19] = −5.65, p<0.001; equilateral triangle vs. leftward arrow, t [19] = −2.90, p = 0.009; hollow diamond vs. cross, t [19] = −3.02, p = 0.007). The results of the simple detection task, in which the subjects were not asked to identify the type of target images, further illustrate that the figures with hole feature were much faster to break suppression. The consistent results of two experiments suggested that hole stimuli may hold a “preference” in the unawareness condition.

## Discussion

In summary, we found that when competing against the same high contrast dynamic noise, the hole stimuli require less time to be detected during the CFS trial than no-hole stimuli, indicating a privileged detection of hole stimuli during the suppression phase of the interocular rivalry.

One might argue that the difference of the similarities in the low-level features (i.e. orientation, spatial frequency) between noise pattern and target images could also contribute to the difference in their suppression times [19]–[23]. Although it's very difficult to manipulate the difference of the similarities between Mondrian pattern and target images, this alternative explanation can be ruled out by controlling the possible low-level feature differences in the stimulus pairs used as target images as far as possible. Indeed, there can be no two geometric figures that differ only in topological properties (i.e., the presence or absence of a hole), without any differences in non-topological factors. Thus, one cannot test for the role of the hole feature in the absence of awareness in complete isolation. We minimized this problem through systematical and careful design of the stimulus pair to prevent subjects from using non-topological properties, including line segments, spatial frequency components, angles, intersections, perimeter length, and the number of edges crossed while scanning a figure, to perform the task. For instance, the ring and S were made to have equal area (and therefore luminous flux), very nearly the same spatial frequency components and perimeter length, and equal averaged edge crossings. The right-angled triangle and the arrow pointing down right were made up of exactly the same three line segments, but they differ in the topological property of holes. The hollow diamond and the cross were designed to orient with their edges parallel to eliminate potential use of orientation cues, and also made to have equal area. P and E were made up of exactly the same five line segments, and designed to excluded the possible use of the local features such as the edge energy and the oriented spatial frequency components. Under such converging operations, these low-level features therefore cannot explain consistently the current finding. The topological account is the only one that explains, in a unified manner across all stimulus pairs used, a privileged detection of “hole”. Thus, the similarities in the low-level features between noise pattern and target images do not appear to be a causal factor in the results presented in this paper.

Based on the current data, we might infer that suppressed figures are processed to the level where the brain can tell a hole stimulus from a no-hole stimulus, suggesting that hole feature can indeed be processed to some extent in the absence of explicit awareness. Previous behavioral and neuroimaging studies have suggested that, the interocular competition occurs at multiple stages along the visual pathway rather than at a single site [24]–[32]. Although rivalry greatly suppresses the activation in the ventral pathway, some information related with suppressed stimulus can indeed arrive at higher brain areas [33], [34]. The anterior temporal lobe (ATL) has been found to be a dedicated region for the processing of topological properties [35]–[37]. Thus, it is possible that, some information related with suppressed stimulus may arrive at the ATL, and then modulates (i.e., enhances) the input signal via feedback projections to help it overcome suppression faster.

How did information arrive at the ATL when figures were suppressed interocularly? One possible account is that, for the hole stimuli, the activity threshold to support awareness is lower than that of the no-hole stimuli, which means that the leaking information of the hole stimuli surviving from incomplete suppression over the multiple stages of rivalry competition may provide relatively more enough information to support awareness. Alternatively, information of the hole feature may reach the ATL via a subcortical pathway which bypassed the cortical site of interocular suppression [38]. Indeed, we found that disruption of the V1 function by using transcranial magnetic stimulation (TMS) has no effect on the detection of the hole stimuli, but significantly impaired the performance of detection no-hole stimuli [39]. However, our behavioral approach cannot distinguish between these two possibilities, further investigation using the neuroimaging techniques is needed to clarify this issue.

Regardless of how the hole information was processed implicitly during the suppressed phase, the present study provides a strong evidence that the hole feature could be processed in the absence of awareness, and there exists a privileged detection of hole stimuli during the suppressed phase in the interocular rivalry.
